# Kyasanur Forest Disease Virus Alkhurma Subtype in Ticks, Najran Province, Saudi Arabia

**DOI:** 10.3201/eid1705.101824

**Published:** 2011-05

**Authors:** Mustafa Mahdi, Bobbie Rae Erickson, J. Andy Comer, Stuart T. Nichol, Pierre E. Rollin, Mohammed A. AlMazroa, Ziad A. Memish

**Affiliations:** Author affiliations: Najran Preventive Medicine Department, Najran, Saudi Arabia (M. Mahdi);; Centers for Disease Control and Prevention, Atlanta, Georgia, USA (B.R. Erickson, J.A. Comer, S.T. Nichol, P.E. Rollin);; Ministry of Health, Riyadh, Saudi Arabia (M.A. AlMazroa, Z.A. Memish)

**Keywords:** viruses, vector-borne infections, tick-borne infections, Kyasanur Forest disease, Alkhurma hemorrhagic fever, Saudi Arabia, letter

**To the Editor:** The lineage of Kyasanur Forest disease virus (KFDV) found in the Kingdom of Saudi Arabia is commonly referred to as Alkhurma hemorrhagic fever virus (AHFV). This virus was first isolated from a specimen collected in 1994 from a butcher living in Makkah Province, who was hospitalized for a hemorrhagic fever from which he died (*1*). The virus was assigned to the genus *Flavivirus* on the basis of reactivity with genus-specific monoclonal antibodies and sequencing of a fragment of the nonstructural 5 (NS5) gene, which showed >89% identity with KFDV. Ten other cases were confirmed among patients who had leukopenia, thrombocytopenia, and elevated liver enzymes. Observations of patients in the original study or in a subsequent analysis (*2*) suggested that Alkhurma hemorrhagic fever (AHF) disease was associated with contact with blood from infected animals, bites from infected ticks, or the drinking of raw milk. However, the exact mode of transmission to humans has still not been fully elucidated. More recently, AHFV RNA was detected in a single pool of sand tampans (*Ornithodoros savignyi*, soft ticks), collected in western Saudi Arabia (*3*), which suggests a link with these ticks.

To analyze the virus association with arthropods further, we collected and identified ticks and mosquitoes in Najran Province, southern Saudi Arabia, during May and June 2009 from different sites close to where human AHF cases had been recently confirmed (*4**,**5*). Camel ticks (*Hyalomma dromedarii*) (130 adults) were collected while they fed on camels, and *O. savignyi* sand tampans (243 adults) were collected from the ground in camel resting places (except 1 collected while feeding on a camel). Mosquitoes were collected by using light traps (203 *Culex decens* females) or as larvae that were then raised in the laboratory (9 *Culiseta* sp. females). Ticks and mosquitoes were stored at room temperature and killed by overnight freezing the day before shipping to the Centers for Disease Control and Prevention (Atlanta, GA, USA). All arthropods were processed in the BioSafety Level 4 laboratory by injecting Vero E6 cells and by intracerebrally inoculating suckling mice with ground pools of either 5 ticks or 10 mosquitoes. All the tick material was used for the tested pools. Isolates of AHFV were obtained from 1 of 13 pools of *H. dromedarii* ticks and 1 of 6 pools of *O. savignyi* sand tampans, both from Al Mishaaliyia district, and from 5 of 8 pools of *O. savignyi* sand tampans from the Al Balad Magan camel market. Virus identity was confirmed by sequencing a 390-nt fragment from the virus core protein C and preM genes. No virus was isolated from any mosquito suspensions.

Phylogenetic analysis of the 7 tick isolates and the available homologous sequences of AHFV are presented in the Figure. The tick AHFV sequences are closely related but distinct from previously reported AHFV sequences from human isolates or from the only sequence reported from ticks collected in 2004 in Jeddah Province. The observed sequences are clustered by site of collection but not by tick species.

In this report, we confirm that the sand tampan (*O. savignyi* tick) is a vector and reservoir of AHFV in Saudi Arabia. Of all arthropods, this tick is one of the most highly adapted to the desert. It can be found in the shade of trees, beside rock fences, on livestock, and in livestock yards, particularly camel yards (*6*). It can feed rapidly during the day or night on camels, goats, sheep, wild mammals, and humans. Sand tampans can survive for long periods without feeding, fulfilling perfectly the role of reservoir for AHFV. This tick has been reported in arid biotopes of northeastern, eastern, and southern Africa (7) and from Arabia to India and Sri Lanka, which suggests a potential wide distribution of AHFV or related viruses. In India, KFDV has been isolated from *Ornithodoros* spp. ticks collected in a bat-inhabited cave (*8*), and experimental transtadial and transovarial transmission of KFDV in *O. crossi* ticks has been reported (*9*).

The isolation of AHFV from the camel tick (*H. dromedarii*) also has public health implications. The capital city of Najran serves as a market for camels and other livestock from Saudi Arabia and Yemen. Adult camel ticks infest mainly camels, and infected ticks can feed on and infect animals just before sale or slaughter. AHF in persons working in the Najran market has been described (5). Unfortunately, no AHFV sequence is available from those cases.

The genetic diversity of the isolated viruses from ticks is quite low. Previous analysis of KFDV and AHFV suggested slow evolution with divergence ≈33 years ago (10). The data reported here clearly strengthen the position of AHFV in the tick-borne flavivirus complex, although the numbers and species of mosquitoes tested were limited. Expanded epidemiologic and molecular studies should provide insight into the distribution and evolution of the virus and identify at-risk regions within Saudi Arabia. Laboratory infection and transmission studies in colonized ticks should clarify the role of *O. savignyi* and *H. dromedarii* ticks in the ecology of AHFV. Currently, public health messages are being developed for the community at risk and local health care workers.

**Figure:**
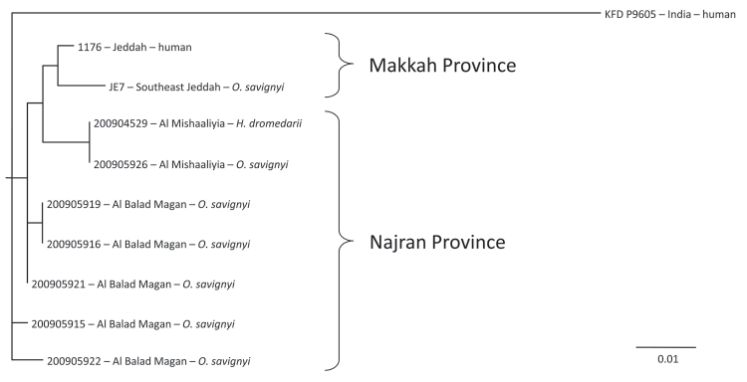
Phylogenetic analysis of Alkhurma viruses isolated from *Ornithodoros savignyi* and *Hyalomma dromedarii* ticks in Najran Province, Kingdom of Saudi Arabia. A 390-bp region of the core protein C and preM genes was amplified and sequenced for each of the isolates (HQ443410–6) by using primers ALK244S (5′-GTGTTGATGCGCATGATGGG-3′) and ALK665R (5′-TGCAGAAACAGTCCACATCA-3′). A maximum-likelihood analysis was conducted with available sequences in GenBank for ALK (NC_004355; *3*) by using Kyasanur Forest disease (AY323490) as the outgroup and the default settings in GARLI version 0.96b8 (www.phylo.org/pdf_docs/zwicklDissertation.pdf). Scale bar indicates substitutions per site.

## References

[1] Zaki AM. Isolation of a flavivirus related to the tick-borne encephalitis complex from human cases in Saudi Arabia. Trans R Soc Trop Med Hyg. 1997;91:179–81. 10.1016/S0035-9203(97)90215-7 9196762

[2] Charrel RN, de Lamballerie X. The Alkhurma virus (family *Flaviviridae*, genus *Flavivirus*): an emerging pathogen responsible for hemorrhage fever in the Middle East [in French]. Med Trop (Mars). 2003;63:296–9. 14579470

[3] Charrel RN, Fagbo S, Moureau G, Alqahtani MH, Temmam S, de Lamballerie X. Alkhurma hemorrhagic fever virus in *Ornithodoros savignyi* ticks. Emerg Infect Dis. 2007;13:153–5. 10.3201/eid1301.061094 17370534 PMC2725816

[4] Memish ZA, Balkhy HH, Francis C, Cunningham G, Hajeer AH, Almuneef MA. Alkhumra haemorrhagic fever: case report and infection control details. Br J Biomed Sci. 2005;62:37–9. 15816213 10.1080/09674845.2005.11978070

[5] Alzahrani AG, Al Shaiban HM, AlMazroa MA, Al-Hayani O, MacNeil A, Rollin PE, Epidemiologic characteristics of Alkhurma hemorrhagic fever in humans in Najran City, Saudi Arabia. Emerg Infect Dis. 2010;16:1882–8. 21122217 10.3201/eid1612.100417 PMC3294564

[6] Hoogstraal H, Wassef HY, Buttiker W. Ticks (Acarina) of Saudi Arabia family *Argasidae*, Ixodidae. In: Wittmer W, Buttiker W, editors. Fauna of Saudi Arabia. Vol. 3. Basel: Ciba Geigy Ltd; 1981. p. 25–110.

[7] Hoogstraal H. African Ixodoidea. I. Ticks of the Sudan (with special reference to Equatorial Province and with preliminary reviews of the genera *Boophilus, Margaropus*, and *Hyalomma*). Washington: Department of the Navy; 1956.

[8] Rajagopalan PK, Paul SD, Sreenivasan MA. Isolation of Kyasanur Forest disease virus from the insectivorous bat, *Rhinolophus rouxi*,and from *Ornithodoros* ticks. Indian J Med Res. 1969;57:805–8. 5820428

[9] Bhat UK, Goverdhan MK. Transmission of Kyasanur Forest disease virus by the soft tick *Ornithodoros crossi.* Acta Virol. 1973;17:337–42. 4148214

[10] Mehla R, Kumar SRP, Yadav P, Barde PV, Yergolkar PN, Erickson BR, Recent ancestry of Kyasanur Forest disease virus. Emerg Infect Dis. 2009;15:1431–7. 10.3201/eid1509.080759 19788811 PMC2819879

